# Treatment of Retained Placenta

**Published:** 1886-06

**Authors:** 


### Treatment of Retained Placenta.

Professor Pajot, in lecture published in the *Gazette des Hopitaux*, gives the following directions:

When the placenta is free, and you are sure of it, you may make very careful traction by the cord. When the placenta is not free, do not use traction by the cord, lest you produce inversion of the uterus or some other fearful accident, such as profuse post partum hemorrhage. Above all, do not give ergot. "*Never give ergot when there is anything in the uterus*," should be your motto. What should you do, then? Place the woman across the bed, follow the cord, and endeavor to detach the placenta and extract it. Then use antiseptic injections, which should be repeated on the following days. If there is still some hemorrhage, give a little ergot; so much for the treatment when the child is born at term. But the treatment may be somewhat different in cases of abortion.

Two cases may present themselves—the one common, the other rare. The common one is the retention of a detached placenta; the rare, the continued growth of an undetached placenta in utero.

In the first case, treat expectantly until there is offensive odor or other sign of putrefaction, but you should immediately interfere by all possible means, not violent, in cases where decomposition has begun. Use the curette and antiseptic injections, for, if you do not remove the after-birth, your woman dies.

In the second case, wait. But leave your patients some tampons, and tell them how they are to be used, lest they die from sudden hemorrhage before the physician arrives.

This is what you should do. What you should *not*, under any circumstances, do is to give ergot when the uterus contains anything, when the placenta is retained, under penalty of committing a homicide, killing the woman; for you can kill her as effectively with ergot under these circumstances as you could by firing a pistol into her breast. When the uterus is empty, however, you may use ergot according to the indications to suppress or prevent hemorrhage.—*L'Union Med. du Canada.*

